# The hidden classroom: how psychosocial infrastructure shapes academic achievement and educational equity in China

**DOI:** 10.3389/fpsyg.2026.1902392

**Published:** 2026-10-07

**Authors:** E. Lin, Kexin Wang, Zhongyu Deng, Junbing Xu

**Affiliations:** 1 School of Education, Shanghai Normal University, Shanghai, China; 2 Principal Training Department, Fujian Institute of Education, Fuzhou, Fujian, China; 3 Newhuadu Business School, Minjiang University, Fuzhou, Fujian, China; 4 School of Education, Central China Normal University, Wuhan, Hubei, China

**Keywords:** academic performance, educational equity, motivational enhancement channel, psychological counseling rooms, psychological well-being channel, psychosocial infrastructure

## Abstract

**Objective:**

While investments in physical school infrastructure are widely recognized as important determinants of student learning, the academic role of psychosocial infrastructure remains insufficiently theorized and empirically underexplored. This study examines whether psychological counseling room infrastructure (PCRI)—defined as the availability and facility condition of school-based psychological counseling rooms—is associated with better students’ academic performance and contributes to educational equity in China.

**Methods:**

Using nationally representative data from the 2014-2015 China Education Panel Survey (CEPS), this study estimates the association between PCRI and students’ academic achievement. PCRI is measured at the school level, while student performance is captured by standardized test scores. The empirical analysis controls for a rich set of student, family, classroom, and school characteristics, and exploits the quasi-random assignment of students to classrooms within schools to reduce selection concerns. Mechanism analyses are conducted to examine the psychological well-being and motivational enhancement channels, and moderation analyses are used to assess whether PCRI has stronger effects among structurally disadvantaged student groups.

**Results:**

PCRI is positively and significantly associated with students’ academic performance. The estimated coefficient is 4.467 with a 95% confidence interval of [3.908, 5.027]. A one-standard-deviation increase in PCRI is associated with a 3.26-point increase in test scores, with a 95% confidence interval of [2.853, 3.670], equivalent to 4.92% of the sample mean. Mechanism analyses reveal two distinct pathways: a psychological well-being channel, reflected in improved mental health and enhanced confidence in the future, and a motivational enhancement channel, reflected in higher educational self-expectations and stronger learning engagement. Moderation analyses further show that the academic returns to PCRI are larger among migrant students, students from less-educated families, students in schools with fewer psychologically trained teachers, and rural students.

**Conclusion:**

The findings suggest that school-based psychological support infrastructure is not merely an auxiliary school resource, but an important component of the educational environment associated with students’ academic achievement and educational equity. By identifying psychological counseling room infrastructure as a distinct school resource, this study highlights how school resources may be linked to academic performance not only through instructional and material conditions, but also through students’ psychological well-being and motivational development.

## 1 Introduction

One of the most enduring questions in education research concerns the productivity of school investments: do additional resources improve what students learn? Scholars have long examined student achievement in relation to family endowments, peer composition, teacher quality, and school resources (Hanushek, 1989, 1997; Hedges et al., 1994). The existing literature has focused predominantly on the academic returns to physical investments—including school construction, campus renovation, technology integration, nutrition programs, and sanitation improvements—documenting measurable gains in student learning and long-run human capital accumulation (Duflo, 2001; Neilson and Zimmerman, 2014; Bianchi et al., 2022; Afridi, 2011; Suryadarma et al., 2006; Kang et al., 2023). Comparatively less attention has been paid to psychosocial infrastructure, which we define as the institutionalized structures and resources through which schools support students’ psychological, emotional, and social functioning. Importantly, psychosocial infrastructure need not be immaterial; rather, it is distinguished from conventional physical resources primarily by its psychosocial function and institutional purpose. Psychological counseling rooms represent one tangible manifestation of such infrastructure by providing dedicated institutional capacity for psychological support. This study therefore examines whether Psychological Counseling Room Infrastructure (PCRI) is associated with students’ academic performance and through what psychological and motivational pathways.

We conceptualize PCRI as a tangible and observable institutional dimension of psychosocial infrastructure, rather than as a direct measure of the broader psychosocial environment or school climate. PCRI captures both the availability and facility condition of school-based psychological counseling rooms and therefore reflects schools’ institutional capacity to provide dedicated psychological support. Drawing on ecological systems theory and school climate theory, we argue that such infrastructure may contribute to a more supportive developmental environment and shape the psychological and motivational conditions under which learning takes place (Bronfenbrenner, 1979; Wang and Degol, 2016; Korpershoek et al., 2020). Specifically, we consider two broad pathways. The psychological well-being channel operates through students’ mental health and confidence in the future, whereas the motivational enhancement channel operates through educational self-expectations and learning engagement. These theories are therefore used to explain the processes through which psychosocial infrastructure may matter for students, rather than to equate PCRI itself with school climate.

Estimating the academic effects of PCRI, however, poses substantial empirical challenges. Schools that invest in more developed psychological counseling infrastructure may differ systematically from those that do not—having stronger leadership, greater organizational capacity, or more positive school climates, all of which may independently contribute to student achievement (Leithwood, 2021). Families may also sort into schools with stronger psychosocial resources, generating selection bias that is particularly pronounced among more advantaged households (Singer, 2025). Reverse causality presents a further concern: schools facing more severe academic or psychological difficulties may be precisely those more likely to establish counseling facilities. These threats to identification make simple comparisons across schools with different levels of PCRI difficult to interpret causally. Compounding these identification challenges, the existing literature on school counseling and school-based mental health services—while documenting benefits for mental health, school climate, disciplinary outcomes, and, in some cases, academic achievement (DiGirolamo et al., 2021; Cipriano et al., 2023; Golberstein et al., 2024)—has largely relied on case studies or small-sample designs that preclude credible causal inference, often lacking adequate control groups or drawing on narrow intervention contexts (Mulhern, 2023). Moreover, this literature concentrates on counseling personnel and targeted service delivery rather than on the institutional availability and facility condition of counseling infrastructure as a school-level resource. The academic consequences of PCRI thus remain insufficiently understood in large-scale education systems.

This study addresses these challenges using data from the 2014-2015 China Education Panel Survey (CEPS), a nationally representative longitudinal survey encompassing approximately 20,000 junior high school students across 112 schools in 28 counties. The CEPS offers two features that are particularly well-suited to our research design. First, it documents substantial variation in PCRI across sampled schools: approximately 46 percent of schools report well-equipped psychological counseling facilities, while the remainder report either no facilities or facilities in need of improvement. This distribution provides sufficient cross-school variation to examine whether differences in PCRI are associated with differences in students’ academic performance. Second, the CEPS explicitly documents the institutional practice of random classroom assignment within schools. In Chinese middle schools, students are commonly assigned to homeroom classes through procedures designed to balance academic ability and demographic characteristics across classrooms. We exploit this naturally occurring assignment as a source of quasi-experimental variation to address the concern that students are systematically sorted across classroom environments according to family background, prior achievement, or motivational traits. In our empirical design, PCRI varies at the school level, while quasi-random classroom assignment strengthens within-school student comparability and mitigates student- and classroom-level selection bias. Our identification strategy thus combines cross-school variation in PCRI with quasi-random allocation of students to classrooms, supplemented by a rich set of individual, household, classroom, and school covariates, as well as district fixed effects and a battery of robustness checks, to provide credible evidence on the academic returns to psychosocial infrastructure investment.

There are four main findings. First, the baseline results show that Psychological Counseling Room Infrastructure (PCRI) is positively and significantly associated with students’ academic performance. Economically, a one-standard-deviation increase in PCRI is associated with a 3.26-point increase in students’ academic performance (4.467 × 0.730), equivalent to approximately 4.92% of the sample mean of SAP (3.261/66.22). Second, the mechanism analysis shows that PCRI operates through two channels: a psychological well-being channel, reflected in the improvement of students’ mental health and the enhancement of their confidence in the future, and a motivational enhancement channel, reflected in the boost of students’ educational self-expectations and the stimulation of learning engagement. Third, the moderation analysis suggests that PCRI plays a stronger compensatory role among students and schools that are more likely to face psychological vulnerability or insufficient psychological support, including migrant students, students from less-educated families, students in schools with fewer mentally trained teachers, and students in rural schools. This finding indicates that PCRI may help disadvantaged students convert institutional psychological support into better academic outcomes.

This study makes three contributions. First, this study broadens the literature on school resources by identifying psychosocial infrastructure as an important but under-examined dimension of the educational environment. Conventional studies emphasize family background, peer composition, teacher quality, material school resources, and school climate, while paying comparatively less attention to institutional structures specifically designed to support students’ psychological and emotional development. We conceptualize psychosocial infrastructure not as the absence of material resources, but as school-based resources distinguished by their psychosocial function and institutional purpose. Within this framework, Psychological Counseling Room Infrastructure (PCRI) represents a tangible and observable dimension of psychosocial infrastructure rather than a direct measure of school climate itself. By focusing on PCRI, this study shows how such infrastructure may be associated with learning through students’ psychological well-being, educational expectations, and learning engagement. This perspective complements growing evidence that social–emotional skills, school climate, and student well-being are important for educational development and academic achievement (Heckman et al., 2006; Jackson, 2018; Kraft, 2019; Cipriano et al., 2023; Algan and Huillery, 2025).

Second, this study extends the literature on student mental health and academic achievement by shifting attention from students’ psychological difficulties to schools’ institutional capacity to provide psychological support. A growing body of research shows that depression, anxiety, psychological distress, and low well-being are associated with weaker academic performance, poorer engagement, and higher dropout risks (Eisenberg et al., 2009; McLeod et al., 2012; Steinmayr et al., 2016). However, much of this evidence comes from particular schools, universities, or localized educational settings. For example, Eisenberg et al. (2009) examine students from a single large public university, while Steinmayr et al. (2016) use a longitudinal sample of 290 German high-school students. Using CEPS data, we examine PCRI as a tangible institutional dimension of psychosocial infrastructure that captures both the availability and facility condition of dedicated psychological counseling spaces. In doing so, we move beyond documenting the association between student mental health and academic outcomes and provide large-scale evidence on the relationship between schools’ psychological-support capacity and students’ academic performance.

Third, this study contributes to the literature on educational inequality by examining the potential compensatory role of school-based psychosocial infrastructure. Prior research suggests that social–emotional factors, school belonging, and school-based mental health services may be particularly important for disadvantaged students, whose educational outcomes are often constrained by psychological stress, weaker emotional support, and limited access to supportive resources (Becker and Luthar, 2002; Durlak et al., 2011; Korpershoek et al., 2020; Graves et al., 2023). Consistent with this literature, our moderation results show stronger associations between PCRI and academic performance among migrant students, students from less-educated families, students in schools with fewer psychologically trained teachers, and rural students. These patterns suggest that the tangible institutional supports represented by PCRI may be especially relevant where alternative sources of psychological and educational support are relatively limited. In this sense, psychosocial infrastructure may have implications not only for student well-being but also for educational equity.

## 2 Theoretical hypotheses

Building on the conceptual foundations developed in the introduction, this section proposes a theoretical framework linking psychological counseling room infrastructure (PCRI) to students’ academic performance. PCRI captures both the availability and facility condition of school-based psychological counseling rooms, and therefore reflects whether schools possess a functional and well-equipped institutional space for providing psychological support. We argue that PCRI may improve students’ academic performance through two theoretically distinct but mutually reinforcing channels: a psychological well-being channel and a motivational enhancement channel. Figure 1 summarizes the hypothesized relationships.

**Figure 1:**
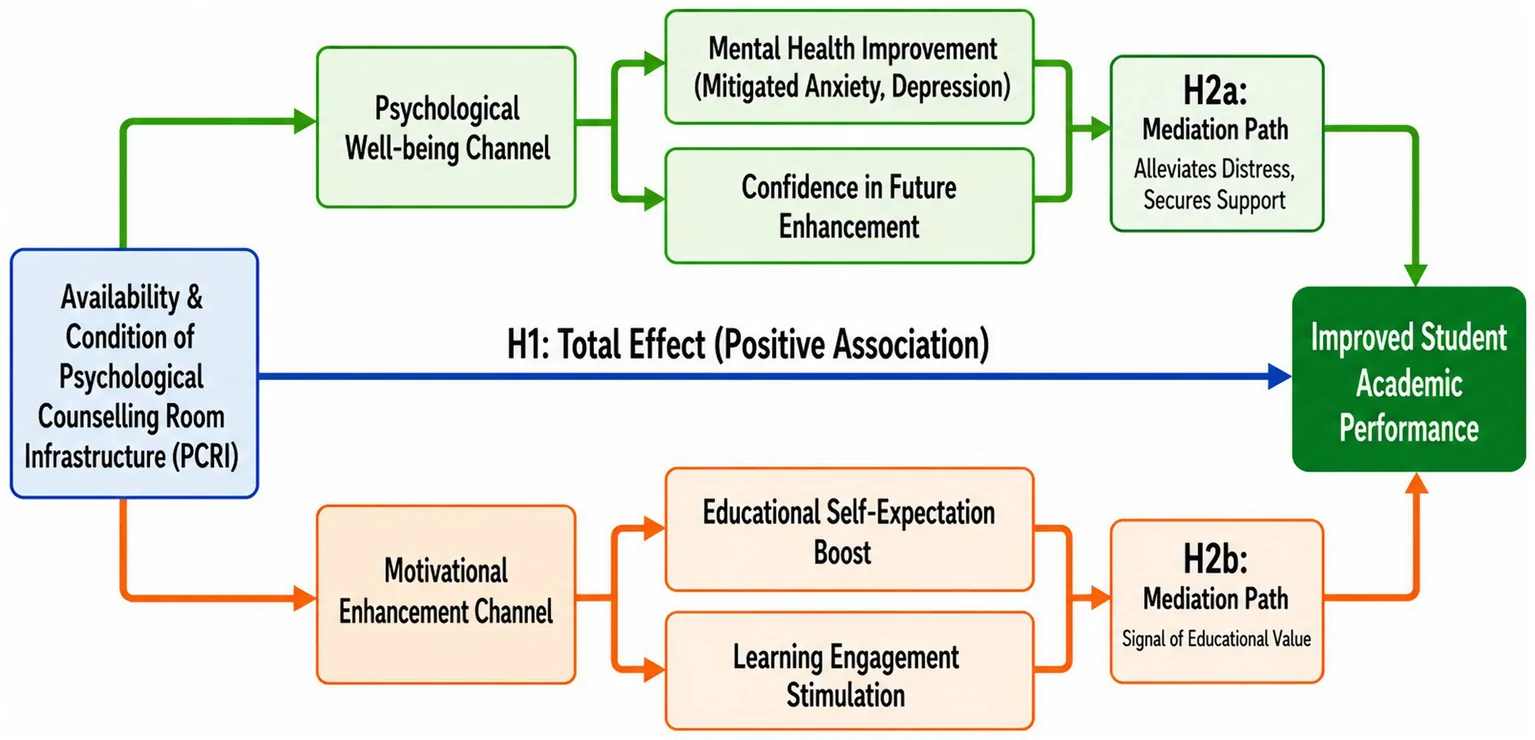
Theoretical hypotheses.

First, PCRI may affect students’ academic performance through the psychological well-being channel, which operates through the improvement of students’ mental health and the enhancement of students’ confidence in the future. Drawing on ecological systems theory and school climate theory, students’ learning outcomes are shaped not only by instructional inputs, but also by the broader emotional and relational environment embedded in schools (Bronfenbrenner, 1979; Wang and Degol, 2016; Korpershoek et al., 2020). Psychological distress, such as anxiety, depressive affect, and emotional instability, can reduce students’ cognitive bandwidth, weaken attention regulation, and undermine their ability to remain engaged in learning tasks (Heckman and Mosso, 2014; Schilbach et al., 2016). By providing an accessible and well-equipped space for counseling, emotional support, and crisis intervention, PCRI may help students regulate negative emotions, alleviate psychological distress, and develop a more secure and supportive perception of the school environment. These processes may improve students’ mental health and enhance their confidence in the future. Better mental health enables students to maintain concentration, regulate emotions, and participate more effectively in classroom activities, while stronger confidence in the future helps students sustain optimism and persistence when facing academic difficulties. Therefore, PCRI may enhance academic performance by strengthening students’ psychological well-being.

Second, PCRI may improve students’ academic performance through the motivational enhancement channel, which operates through the boost of students’ educational self-expectations and the stimulation of learning engagement. According to Expectancy-Value Theory, students’ academic effort depends on their expectations for success and the value they attach to educational achievement (Wigfield and Eccles, 2000; Eccles and Wigfield, 2002). Self-Determination Theory further emphasizes that supportive school environments can strengthen students’ perceived competence, autonomy, and intrinsic motivation (Deci and Ryan, 2000). In this sense, visible and functional investment in psychological counseling room infrastructure may communicate an institutional signal that students are valued, supported, and capable of future development. This signal may boost students’ educational self-expectations by encouraging them to set higher academic goals and imagine more promising educational trajectories. It may also stimulate learning engagement by reinforcing students’ perceived competence, emotional security, and willingness to invest effort in learning. Students with higher educational self-expectations are more likely to set ambitious academic goals, while students with stronger intrinsic motivation are more likely to devote time and effort to academic tasks, complete assignments, and persist despite setbacks. Accordingly, PCRI may contribute to academic performance not only by alleviating psychological difficulties, but also by cultivating future-oriented beliefs and motivational resources that support sustained learning. Based on the above discussion, we propose the following hypotheses:

*H1*: Psychological counseling room infrastructure is positively associated with students’ academic performance.

*H2a*: PCRI is associated with better students’ academic performance through the psychological well-being channel, as reflected in the improvement of students’ mental health and the enhancement of students’ confidence in the future.

*H2b*: PCRI is associated with better students’ academic performance through the motivational enhancement channel, as reflected in the boost of students’ educational self-expectations and the stimulation of learning engagement.

## 3 Research design

### 3.1 Sample selection and data source

We use data from the 2014-2015 China Education Panel Survey (CEPS) conducted by the China Survey and Data Center of Renmin University of China. CEPS established the academic year of 2013–2014 as its baseline, and initiated the survey with two concurrent cohorts: first-year junior high school students (grade 7) and third-year junior high school students (grade 9). Using the average educational attainment of the population and the proportion of the migrant population as stratification criteria, the CEPS randomly selects 28 county-level units—comprising counties, districts, and cities—across the nation to serve as the survey locations. The survey was conducted school-by-school, with a random selection of 112 schools and 438 classes within the designated county-level units. Every student in the chosen classes was included in the sample, yielding in a baseline survey that encompassed approximately 20,000 students. During the 2014-2015 academic year, the survey successfully followed up with the seventh-grade students from the 2013 baseline cohort. Utilizing the codes for student, class, and school provided in the survey, we match and consolidates data on middle school students, their respective classes, principal details, and parental information. Following Xu and Li (2018), we use specific questions from the CEPS questionnaire to verify whether students were randomly assigned to classes within schools, restricting our sample to eighth-grade students in schools where random assignment was confirmed. This restriction is motivated by a specific endogeneity concern: if unobserved family characteristics—such as parental educational philosophies—systematically influence both class placement and student academic outcomes, and if these characteristics are correlated with the presence of school-based counseling rooms, naive estimates would be confounded. Restricting the analysis to schools implementing random classroom assignment mitigates self-selection bias at the intra-school level, ensuring that observed differences in outcomes across students within the same school reflect treatment variation rather than compositional sorting. Our final sample comprises 6,055 eighth-grade students across 231 classes in 88 schools. Appendix Table A1 documents the detailed sample-construction process. Because restricting the analysis to schools with confirmed random classroom assignment may generate sample-selection concerns, we further compare the restricted sample with otherwise eligible CEPS observations across key observable characteristics. As reported in Appendix Table A2, the two samples are similar in several dimensions, including school location, class size, homeroom-teacher gender, and migrant status. However, some differences are observed in teacher characteristics, school resources, and selected family characteristics. We therefore do not interpret the restricted sample as fully representative of the broader eligible CEPS sample and explicitly account for this concern in additional sample-selection robustness analyses.

### 3.2 Research model

Referring to Lipscomb (2007), we employ a fixed effects model to estimate whether access to PCRI affects students’ academic performance. The specific model is as follows:

$${\mathrm{SAP}}_{i}=\alpha_{0}+\beta {\text{PCRI}}_{s}+\alpha_{1}\sum {\text{Control}}_{i,h,t,s}+\sigma_{d}+\varepsilon_{i,h,t,s}$$ (1)

where *i* denotes student, *t* denotes homeroom teacher, *h* denotes household, *s* denotes school, and *d* denotes district. The outcome variable, students’ academic performance (SAP), is measured as the average score across three core subjects: Chinese, Mathematics, and English. The key independent variable, PCRI, is a school-level ordinal measure constructed from the CEPS 2014-2015 principal questionnaire, taking a value of 0 if the school has no psychological counseling room, 1 if it has a room with facilities needing improvement, and 2 if it has a room with good facilities. Higher values thus reflect more developed psychological counseling infrastructure in terms of both availability and facility condition. β is the coefficient of interest, capturing the association between PCRI and students’ academic performance. A positive and statistically significant estimate of β would indicate that students in schools with more developed psychological counseling infrastructure achieve higher academic performance, ceteris paribus. Control denotes a vector of control variables spanning four levels: individual, household, class, and school. Additionally, our paper also includes district fixed effects ( $\sigma_{d}$ ) to absorb time-invariant differences across districts—including variation in local economic development, educational resources, teacher qualifications, and examination standards—that might otherwise confound the estimates. Finally, this paper clusters the standard errors of coefficients within individuals to mitigate serial autocorrelation of residuals within individuals.

### 3.3 Variables definitions

#### 3.3.1 Independent variable: psychological counseling room infrastructure (PCRI)

The key independent variable in this study is Psychological Counseling Room Infrastructure (PCRI), which captures both the availability and facility condition of school-based psychological counseling rooms. In the CEPS 2014-2015 principal questionnaire, school principals were asked: “Does the school have a psychological counseling room?”. The response options were: “No,” “Yes, but the facilities should be improved”, and “Yes, and the facilities are good”. Specifically, schools that reported having no psychological counseling room are assigned a value of 0; schools that reported having a psychological counseling room but with facilities needing improvement are assigned a value of 1; and schools that reported having a psychological counseling room with good facilities are assigned a value of 2. Therefore, higher values of PCRI indicate more developed psychological counseling room infrastructure, reflecting both greater access to school-based psychological support facilities and better facility conditions. This coding strategy is consistent with Xia et al. (2020), who use CEPS data and code school playground facilities as 0 for no playground, 1 for having a playground but with equipment needing improvement, and 2 for having a playground with good equipment. Schools with missing responses to this questionnaire item are excluded from the analysis. Importantly, PCRI captures the availability and principal-reported physical condition of counseling-room infrastructure, rather than the realized quality or utilization of psychological services. It does not directly measure counselor qualifications, staffing intensity, frequency of use, documented service hours, or student satisfaction.

To assess whether our findings are sensitive to this ordinal measurement strategy, we further conduct a robustness check using an alternative binary measure for availability of psychological counseling room infrastructure. In this specification, schools without a psychological counseling room infrastructure are assigned a value of 0, while schools that reported having a psychological counseling room infrastructure are assigned a value of 1, regardless of whether the facilities need improvement or are in good condition. The results remain substantively unchanged, suggesting that our main findings are not driven by the specific ordinal coding of the PCRI variable.

#### 3.3.2 Dependent variable: students’ academic performance (SAP)

The dependent variable in this study is students’ academic performance (SAP). Following Xie and He (2023) and Qin et al. (2025), we measure SAP using students’ midterm examination scores provided directly by the surveyed schools. Compared with self-reported academic performance, school-reported examination records help reduce measurement error and reporting bias. Specifically, the CEPS questionnaire reports students’ raw midterm examination scores in three core subjects: Chinese, mathematics, and English. To ensure comparability across subjects, we first convert the raw scores of each subject to a 100-point scale. We then calculate the average score across the three subjects and use this composite measure as the baseline indicator of students’ academic performance. Formally, SAP is defined as the mean of the standardized Chinese, Mathematics, and English midterm scores, with higher values indicating better academic performance.

One potential concern is that school-based examination scores may not be fully comparable across schools because test difficulty, grading standards, and curriculum progress may vary. To address this concern, we further use students’ nationally comparable cognitive ability test scores administered uniformly by CEPS at the time of the survey as an alternative measure of academic performance in the robustness checks. The results remain substantively unchanged, suggesting that our core findings are not driven by cross-school differences in examination standards.

#### 3.3.3 Control variables

Referring to Wei et al. (2010) and Liu et al. (2026), we include a rich set of control variables to account for observable differences in student background, family environment, classroom characteristics, teacher attributes, and school conditions. This specification is consistent with the view that student achievement is jointly shaped by prior achievement, family inputs, school inputs, and teacher-related factors. Specifically, we control for four groups of covariates. First, student-level controls include gender (Gender), prior academic achievement measured by last year’s midterm examination results (PriorScore), internet access at home (InternetAccess), physical disability status (Disability), and the number of close friends (NumFriends). Second, family-level controls include family economic status (FamilyEco), residential community environment (CommunityEnv), living arrangement (LivingArrangement), parent–child relationship (ParentChildRel), migrant status (Migrant), and parental supervision (ParentalSupervision). Third, classroom and homeroom-teacher controls include class size (ClassSize), homeroom teacher gender (HT_Gender), homeroom teacher teaching experience (HT_TeachingExp), and whether the homeroom teacher has received psychological training (HT_PsychTraining). Fourth, school-level controls include school location (SchoolLocation), public school status (PublicSchool), student-teacher ratio (StudentTeacherRatio), and principal tenure at the school (PrincipalTenure). Appendix Table A3 represents variable definitions.

### 3.4 Descriptive statistics

Table 1 reports the descriptive statistics for the main variables used in the empirical analysis. The sample includes 6,055 student-level observations, 231 classroom-level observations, and 88 school-level observations. The dependent variable, students’ academic performance (SAP), has a mean value of 66.22 and a standard deviation of 19.22, with a minimum value of 1.667 and a maximum value of 99. This indicates that the average student performance is above the midpoint of the 100-point scale, while the relatively large standard deviation suggests substantial heterogeneity in academic achievement across students. The key independent variable, psychological counseling room infrastructure (PCRI), has a mean value of 1.295, a standard deviation of 0.730, and ranges from 0 to 2. Since PCRI is coded as 0 for schools without a psychological counseling room, 1 for schools with a psychological counseling room whose facilities need improvement, and 2 for schools with a psychological counseling room in good condition, the average value suggests that many schools in the sample have established psychological counseling rooms, but the quality of this infrastructure varies across schools. In other words, the descriptive statistics show meaningful variation in school-based psychological counseling infrastructure, which provides empirical variation for examining its relationship with student outcomes.

**Table 1:** Descriptive statistics of main variables.

Variables	Observation	Mean	SD	Min	Max
Student characteristics					
SAP	6,055	66.22	19.22	1.667	99
Gender	6,055	0.498	0.500	0	1
PriorScore	6,055	70.78	8.196	18.50	97.72
InternetAccess	6,055	0.648	0.478	0	1
Disability	6,055	0.0390	0.194	0	1
NumFriends	6,055	2.093	0.803	0	4.605
Family and parent characteristics					
FamilyEco	6,055	2.823	0.612	1	5
CommunityEnv	6,055	0.892	0.310	0	1
LivingArrangement	6,055	0.685	0.465	0	1
ParentChildRel	6,055	0.513	0.500	0	1
Migrant	6,055	0.184	0.387	0	1
ParentalSupervision	6,055	13.84	2.388	6	18
Classroom and teacher characteristics					
ClassSize	231	3.855	0.279	2.773	4.477
HT_Gender	231	0.636	0.482	0	1
HT_TeachingExp	231	14.60	8.573	0	50
HT_PsychTraining	231	0.199	0.400	0	1
School characteristics					
PCRI	88	1.295	0.730	0	2
SchoolLocation	88	0.455	0.501	0	1
PublicSchool	88	0.909	0.289	0	1
StudentTeacherRatio	88	12.90	4.783	3	30.80
PrincipalTenure	88	11.44	6.740	1	30

## 4 Results

### 4.1 Baseline results

In this section, we examine whether psychological counseling room infrastructure (PCRI) is associated with students’ academic performance (SAP). Table 2 reports the baseline regression results. We adopt a stepwise specification strategy. Column (1) includes county fixed effects only, while column (2) further adds student-, family-, classroom/teacher-, and school-level control variables. The coefficient on PCRI is positive and statistically significant at the 1% level across both specifications. In column (1), the estimated coefficient is 4.721 (95% CI [3.908, 5.027]), indicating a positive association between psychological counseling room infrastructure and students’ academic performance after controlling for county fixed effects. After adding the full set of control variables in column (2), the coefficient remains positive and significant, with an estimate of 4.467. This suggests that schools with more developed psychological counseling room infrastructure are associated with higher students’ academic performance. In terms of magnitude, the estimate from our preferred specification is substantively meaningful. Given that the standard deviation of PCRI is 0.730, a one-standard-deviation increase in psychological counseling room infrastructure is associated with a 3.26-point (95% CI [2.853, 3.670]) increase in students’ academic performance (4.467 × 0.730). This effect corresponds to approximately 4.92% of the sample mean of SAP (3.261/66.22). Relative to the sample standard deviation of SAP, this corresponds to an effect size of approximately 0.17 standard deviations. Thus, the estimated association is not only statistically significant but also educationally meaningful, suggesting that better-developed psychological counseling room infrastructure is linked to higher levels of student academic achievement.

**Table 2:** Psychological counseling room infrastructure and students’ academic performance.

Variables	Students’ academic performance: SAP	
	(1)	(2)
PCRI	4.721*** (10.648)	4.467*** (15.653)
Control vars	No	Yes
County FE	Yes	Yes
Observations	6,055	6,055
Adj_*R*^2^	0.225	0.730

*** denotes significance at 1%, ** at 5%, and * at 10%. County FE is county fixed effects. Control Vars include student-level controls (Gender, PriorScore, InternetAccess, Disability, NumFriends), family-level controls (FamilyEco, CommunityEnv, LivingArrangement, ParentChildRel, Migrant, ParentalSupervision), classroom and homeroom-teacher controls (ClassSize, HT_Gender, HT_TeachingExp, HT_PsychTraining), and school-level controls (SchoolLocation, PublicSchool, StudentTeacherRatio, PrincipalTenure).

### 4.2 Robust checks

#### 4.2.1 Instrumental variable (IV) results

A potential concern is that the association between psychological counseling room infrastructure (PCRI) and students’ academic performance (SAP) may reflect reverse causality or unobserved school characteristics. To address this concern, we employ an instrumental-variable strategy based on a leave-one-out measure of local psychological-support infrastructure. Following Mendolia et al. (2018), Fletcher et al. (2020), and Norris (2023), we construct IV_PCRI as the average PCRI of other schools in the same city, excluding the focal school. Schools within the same city are subject to common educational governance, fiscal capacity, and policy implementation, generating substantial local co-movement in psychological-support infrastructure. The exclusion restriction requires that, conditional on the rich set of student-, family-, classroom/teacher-, and school-level covariates and county fixed effects, other schools’ PCRI does not independently affect the focal student’s academic performance. Although the leave-one-out construction eliminates mechanical own-school correlation, shared local educational priorities or resource conditions may still generate residual correlation between the instrument and student outcomes.

The first-stage results indicate a strong relationship between IV_PCRI and PCRI, as illustrated in Appendix Figure A1. The Kleibergen–Paap Wald F-statistic is 4,537, substantially exceeding the Stock–Yogo critical value of 16.38, suggesting that weak identification is unlikely to be a concern (Kleibergen and Paap, 2006; Stock and Yogo, 2005). As reported in Column (1) of Table 3, the second-stage coefficient on PCRI remains positive and statistically significant, consistent with the baseline results.

**Table 3:** Robust check.

Variables	SAP	CAT	Students’ academic performance: SAP		
	(1)	(2)	(3)	(4)	(5)
PCRI	6.348*** (19.030)	0.137*** (8.574)		4.444*** (15.710)	4.467*** (4.623)
PCRI_2			3.849*** (6.665)		
IV	Yes				
Alternative measurement		Yes	Yes		
Exclude the Influence of extreme SAP				Yes	
Exclude the influence of within-school error correlation					Yes
Control vars	Yes	Yes	Yes	Yes	Yes
County FE	Yes	Yes	Yes	Yes	Yes
Observations	6,028	6,012	6,055	6,055	6,055
Adj_*R*^2^	0.728	0.463	0.719	0.729	0.730

*** denotes significance at 1%, ** at 5%, and * at 10%. County FE is county fixed effects. Control Vars include student-level controls (Gender, PriorScore, InternetAccess, Disability, NumFriends), family-level controls (FamilyEco, CommunityEnv, LivingArrangement, ParentChildRel, Migrant, ParentalSupervision), classroom and homeroom-teacher controls (ClassSize, HT_Gender, HT_TeachingExp, HT_PsychTraining), and school-level controls (SchoolLocation, PublicSchool, StudentTeacherRatio, PrincipalTenure).

Overall, the IV estimates provide suggestive evidence that the positive relationship between PCRI and student academic performance is not entirely attributable to the endogeneity concerns considered here. However, because the exclusion restriction cannot be directly tested and may be affected by shared local institutional factors, we do not interpret the IV estimates as definitive causal evidence.

#### 4.2.2 Alternative measurement for students’ academic performance

One concern is that school-reported midterm scores may not be fully comparable across schools because test difficulty, grading standards, and curriculum progress may vary across schools. To address this issue, we follow Yang (2025) and use the nationally standardized Cognitive Ability Test (CAT) score administered by the CEPS survey team as an alternative outcome measure. Because the CAT was uniformly administered to all sampled students, it provides a comparable measure of students’ cognitive ability across schools. As reported in Column (2) of Table 3, the coefficient on PCRI remains positive and statistically significant when CAT is used as the dependent variable. This finding indicates that our main conclusion is not driven by cross-school differences in school-based examinations and that better-developed psychological counseling room infrastructure is positively associated with students’ standardized cognitive performance.

We further examine whether the results are sensitive to the construction of the baseline SAP measure. First, we standardize Chinese, mathematics, and English scores separately and construct an alternative academic-performance measure as the mean of the three subject-specific *z*-scores. This specification reduces concerns that differences in score distributions across subjects mechanically influence the composite measure. Second, we apply principal component analysis (PCA) to the three subject scores and use the first principal component as an alternative outcome, thereby allowing the relative contribution of each subject to be determined by their covariance structure rather than imposing equal weights. The three subject scores exhibit good internal consistency (Cronbach’s *α* = 0.8618), and the first principal component explains 80.57% of their total variance. As reported in Appendix Table A4, the coefficient on PCRI remains positive and statistically significant under both alternative constructions. These results indicate that the baseline finding is not driven by the particular aggregation method used to construct SAP.

#### 4.2.3 Alternative measurement for psychological counseling room infrastructure (PCRI_2)

To examine whether our results are sensitive to the ordinal coding of PCRI, we construct an alternative binary measure, PCRI_2, that captures the availability of a psychological counseling room. Specifically, PCRI_2 equals 1 if the school reports having a psychological counseling room, regardless of whether the facilities need improvement or are in good condition, and 0 otherwise. This measure abstracts from facility quality and focuses only on whether psychological counseling room infrastructure is present. As reported in Column (3) of Table 3, the coefficient on PCRI_2 remains positive and statistically significant at the 1% level. This finding indicates that our main result is not driven by the specific ordinal coding of PCRI and remains robust when psychological counseling room infrastructure is measured as a binary availability indicator.

We further estimate a fully categorical specification that relaxes the equal-spacing assumption implicit in the ordinal PCRI measure. Specifically, we define PCRI_medium as an indicator for schools with a psychological counseling room whose facilities require improvement (PCRI = 1) and PCRI_good as an indicator for schools with a well-equipped counseling room (PCRI = 2), with schools without a counseling room (PCRI = 0) serving as the reference category. As reported in Appendix Table A5, the coefficients on PCRI_medium and PCRI_good are 1.312 and 7.628, respectively, and both are statistically significant. Moreover, the difference between the two coefficients is 6.315 and statistically significant at the 1% level. These results indicate that the positive association between PCRI and academic performance is not driven by the linear ordinal coding of the baseline measure and is more pronounced among schools reporting better-equipped counseling facilities.

#### 4.2.4 Excluding the influence of extreme academic performance

To rule out the possibility that our baseline findings are driven by unusually low or high values of students’ academic performance, we winsorize SAP at the 1st and 99th percentiles and re-estimate the baseline model. This approach is consistent with prior CEPS-based studies that apply winsorization to test scores or other continuous student-level outcomes to reduce the influence of extreme observations (Xie and He, 2023). In our setting, winsorizing SAP limits the influence of extreme examination scores while retaining the original estimation sample. As reported in Column (4) of Table 3, the coefficient on PCRI remains positive and statistically significant after winsorizing SAP. This finding indicates that the estimated association between psychological counseling room infrastructure and students’ academic performance is not driven by students with unusually low or high test scores. Rather, the positive relationship between PCRI and SAP remains robust after accounting for the potential influence of extreme academic performance.

#### 4.2.5 Excluding the influence of within-school error correlation

To rule out the possibility that our statistical inference is affected by correlated error terms among students within the same school, we re-estimate the baseline model using school-level clustered standard errors. This adjustment is important because CEPS has a nested data structure in which students are grouped within schools. Students from the same school may share common teachers, school management practices, peer environments, and other unobserved school-level factors, which can generate within-school correlation in the error terms. Ignoring such correlation may lead to underestimated standard errors and overstated statistical significance. Following the cluster-robust inference literature, we therefore cluster standard errors at the school level, allowing for arbitrary correlation among students within the same school while assuming independence across schools (Cameron and Miller, 2015; Abadie et al., 2023). As reported in Column (5) of Table 3, the coefficient on PCRI remains positive and statistically significant after clustering standard errors at the school level. This finding indicates that the statistical significance of our main result is not driven by the assumption of independent errors across students. In other words, the positive association between psychological counseling room infrastructure and students’ academic performance remains robust after accounting for within-school error correlation.

#### 4.2.6 Placebo tests

To assess whether the baseline association could arise from chance treatment assignment, we conduct a placebo test following the logic of randomization inference (Keng, 2018). We randomly assign a pseudo-treatment indicator, PCRI^Placebo^, to the same number of schools as those classified as having well-equipped psychological counseling rooms and re-estimate Equation 1. Repeating this procedure 500 times yields the empirical distribution of placebo coefficients shown in Figure 2. The placebo estimates are tightly centered around zero, whereas the baseline PCRI coefficient of 4.467 lies in the far right tail of the distribution. This pattern indicates that randomly generated treatment assignments rarely produce estimates comparable to the observed association. Accordingly, the placebo exercise provides a falsification check against chance assignment patterns, while not addressing potential school-level sorting or other unobserved confounding.

**Figure 2:**
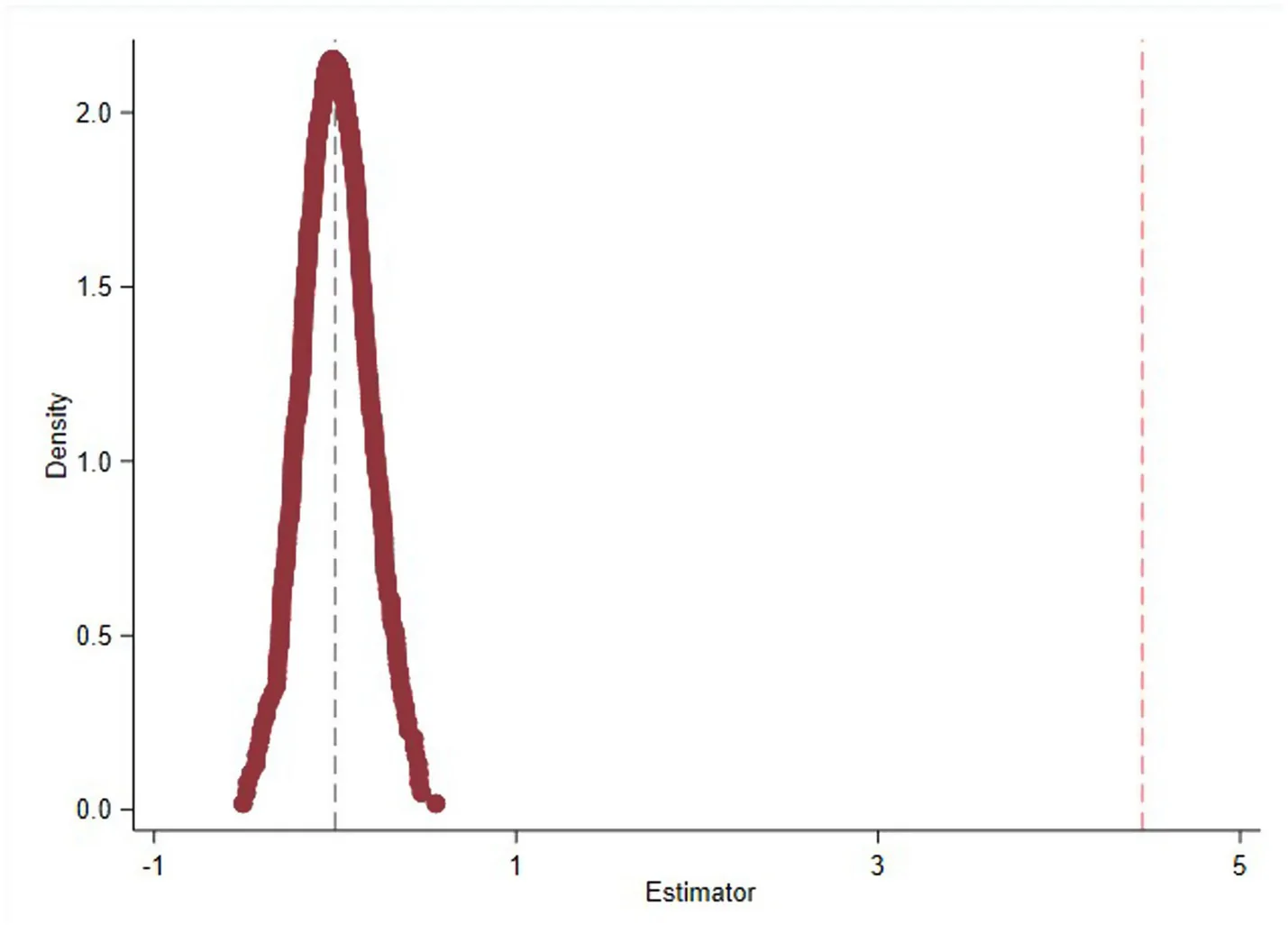
Placebo test.

#### 4.2.7 Sensitivity to omitted-variable bias

We further assess the sensitivity of the baseline estimate to omitted-variable bias using the coefficient-stability approach of Oster. As reported in Appendix Table A7, the coefficient on PCRI is 4.738 when controlling for individual-level characteristics and declines only modestly to 4.467 after additionally accounting for family-, classroom/teacher-, and school-level covariates. Meanwhile, *R*^2^ increases from 0.677 to 0.730. The corresponding Oster statistics yield *δ* values ranging from 1.763 to 1.867, indicating that selection on unobservables would need to be approximately 1.8 times as strong as selection on observables to explain away the estimated association. These results suggest substantial stability of the baseline estimate to potential omitted-variable bias.

#### 4.2.8 Robustness to sample selection

Because the baseline analysis is restricted to schools with confirmed random classroom assignment, we further assess the sensitivity of the estimates to observable sample selection using inverse-probability-of-selection weighting (IPSW). The weighting procedure reweights observations in the analytical sample by the inverse of their estimated probability of inclusion. As reported in Appendix Table A7, the PCRI coefficient declines only modestly from 4.467 to 4.260 after reweighting and remains statistically significant at the 1% level. The similarity of the weighted and unweighted estimates indicates that observable sample-selection differences associated with the random-assignment restriction are unlikely to account for the baseline association.

### 4.3 Channel analysis

To further examine the potential channels through which psychological counseling room infrastructure (PCRI) is associated with students’ academic performance, we focus on two broad pathways: a psychological well-being channel and a motivational enhancement channel. The former is captured by students’ mental health and confidence in the future, while the latter is reflected in students’ educational self-expectations and learning engagement. In addition to examining the relationship between PCRI and each mediator, we conduct formal mediation tests using the product-of-coefficients approach. Tables 4 and 5 report the total, direct, and indirect effects, together with the proportion mediated, Sobel statistics, and bootstrapped confidence intervals.

**Table 4:** Psychological well-being channel.

Variables	(1)	(2)	(3)	(4)	(5)	(6)
	SAP	SMH	SAP	SAP	FC	SAP
PCRI	4.467*** (15.653)	1.473*** (3.123)	4.421*** (15.496)	4.435*** (15.434)	0.030* (1.765)	4.381*** (15.337)
SMH			0.032*** (4.688)			
FC						1.802*** (9.039)
Control vars	Yes	Yes	Yes	Yes	Yes	Yes
County FE	Yes	Yes	Yes	Yes	Yes	Yes
Observations	6,055	6,055	6,055	5,977	5,977	5,977
Adj_*R*^2^	0.730	0.187	0.735	0.731	0.117	0.747
Indirect effect	0.047			0.054		
Sobel *z*	2.599**			1.732*		
Bootstrap 95% CI	[0.017, 0.076]			[−0.004, 0.112]		

*** denotes significance at 1%, ** at 5%, and * at 10%. County FE is county fixed effects. Control Vars include student-level controls (Gender, PriorScore, InternetAccess, Disability, NumFriends), family-level controls (FamilyEco, CommunityEnv, LivingArrangement, ParentChildRel, Migrant, ParentalSupervision), classroom and homeroom-teacher controls (ClassSize, HT_Gender, HT_TeachingExp, HT_PsychTraining), and school-level controls (SchoolLocation, PublicSchool, StudentTeacherRatio, PrincipalTenure).

**Table 5:** Motivational enhancement channel.

Variables	(1)	(2)	(3)	(4)	(5)	(6)
	SAP	SEE	SAP	SAP	LE	SAP
PCRI	4.467*** (15.653)	0.389*** (4.599)	4.159*** (15.006)	4.469*** (15.502)	0.089** (2.223)	4.432*** (15.382)
SEE			0.794*** (17.785)			
LE						0.412*** (4.930)
Control vars	Yes	Yes	Yes	Yes	Yes	Yes
County FE	Yes	Yes	Yes	Yes	Yes	Yes
Observations	6,055	6,055	6,055	5,981	5,981	5,981
Adj_*R*^2^	0.730	0.206	0.731	0.730	0.148	0.730
Indirect effect	0.309			0.037		
Sobel *z*	4.453***			2.026**		
Bootstrap 95% CI	[0.158, 0.459]			[0.002, 0.072]		

*** denotes significance at 1%, ** at 5%, and * at 10%. County FE is county fixed effects. Control Vars include student-level controls (Gender, PriorScore, InternetAccess, Disability, NumFriends), family-level controls (FamilyEco, CommunityEnv, LivingArrangement, ParentChildRel, Migrant, ParentalSupervision), classroom and homeroom-teacher controls (ClassSize, HT_Gender, HT_TeachingExp, HT_PsychTraining), and school-level controls (SchoolLocation, PublicSchool, StudentTeacherRatio, PrincipalTenure).

#### 4.3.1 Psychological well-being channel

We first examine whether better psychological counseling room infrastructure supports the psychological well-being channel through the improvement of students’ mental health and the enhancement of students’ confidence in the future. Specifically, we focus on two outcomes: students’ mental health and their confidence in the future. Following Yang (2024), we construct a composite measure of students’ mental health (SMH) using students’ responses to the CEPS question: “In the past seven days, have you had the following feelings?”. The questionnaire covers 10 dimensions of students’ emotional and psychological states^1^. Responses are reported on a five-point scale ranging from 1 (“Never”) to 5 (“Always”). To facilitate interpretation, these items are reverse-coded where necessary so that higher values consistently indicate better mental health. Following prior educational research that constructs composite indices from multiple student questionnaire items (Wang and Degol, 2016; West et al., 2016), we apply principal component analysis to these items and use the first principal component to construct the SMH index. The ten items exhibit high internal consistency, with a Cronbach’s *α* of 0.9128, and the first principal component explains 57.36% of the total variance. As reported in Column (1)–(3) of Table 4, PCRI is positively associated with students’ mental health. The indirect effect through SMH is 0.047 and direct effect of PCRI on SAP is 4.421, with a Sobel statistic of 2.599 and a bootstrapped 95% confidence interval of [0.017, 0.076]. These results are consistent with the view that better psychological counseling room infrastructure is associated with academic performance partly through better student mental health. This result is consistent with the argument that well-equipped psychological counseling rooms may contribute to the improvement of students’ mental health by providing more accessible psychological support, alleviating negative emotions, improving emotional adjustment, and reducing psychological barriers to learning.

As a second dimension of the psychological well-being channel, we examine the enhancement of students’ confidence in the future. Following Wang et al. (2022), confidence in the future (FC) is constructed based on the CEPS question: “Do you have confidence in your future?” Responses are measured on a four-point Likert scale, ranging from 1 (“No confidence at all”) to 4 (“Very confident”), with higher values indicating greater confidence. As shown in Column (2), the coefficient on PCRI is positive and statistically significant at the 10% level. The indirect effect through FC is 0.054 and direct effect of PCRI on SAP is 4.381, and is marginally significant according to the Sobel test (z = 1.732) and a bootstrapped 95% confidence interval of [−0.004, 0.112]. This result indicates that better psychological counseling room infrastructure is associated with stronger future confidence among students. One possible explanation is that adequately equipped counseling rooms create a more supportive school environment, which may help students develop more positive expectations about their long-term academic and personal development. Taken together, Table 4 provides suggestive evidence that PCRI operates through the psychological well-being channel by promoting the improvement of students’ mental health and the enhancement of students’ confidence in the future.

#### 4.3.2 Motivational enhancement channel

Second, we examine whether PCRI supports the motivational enhancement channel through the boost of students’ educational self-expectations and the stimulation of learning engagement (LE). Specifically, we focus on two dimensions: students’ educational self-expectations and learning motivation. Following Peng et al. (2025), students’ educational self-expectations (SEE) are measured using the CEPS question: “What level of education do you expect to complete?” Responses are converted into the corresponding years of schooling typically required to complete each educational level. Higher values therefore indicate stronger educational aspirations. Columns (1)–(3) of Table 5 show that the coefficient on PCRI is positive and statistically significant. The corresponding indirect effect is 0.309 and direct effect of PCRI on SAP is 4.159, with a Sobel statistic of 4.453 and a bootstrapped 95% confidence interval of [0.158, 0.459]. This result suggests that students in schools with better psychological counseling room infrastructure tend to hold higher educational expectations. This finding is consistent with the view that improved school-based psychological support may contribute to the boost of students’ educational self-expectations by helping students develop more positive self-perceptions, stronger future-oriented beliefs, and higher educational aspirations (Ryan and Deci, 2000).

Finally, we examine the stimulation of learning engagement (LE). Learning engagement (LE) is measured by the average amount of time students spend per day in a typical week attending class-related tutoring or counseling sessions and completing homework assigned by teachers and parents, with higher values indicating stronger engagement in learning-related activities. As reported in Column (4)–(6), the coefficient on PCRI is positive and statistically significant. The indirect effect through learning engagement is 0.037 and direct effect of PCRI on SAP is 4.432, with a Sobel statistic of 2.026 and a bootstrapped 95% confidence interval of [0.002, 0.072]. This result suggests that better psychological counseling room infrastructure is associated with stronger learning motivation. In other words, well-equipped psychological counseling rooms may not only improve students’ emotional adjustment, but also stimulate intrinsic learning motivation by encouraging stronger academic engagement, persistence, and willingness to invest effort in learning.

Overall, the results in Tables 4 and 5 support the proposed mechanism framework. PCRI is associated with students’ academic performance through both the psychological well-being and motivational enhancement channels. Within the psychological well-being channel, the evidence is stronger for mental health than for confidence in the future. Within the motivational enhancement channel, educational self-expectations account for the largest indirect effect, while learning engagement provides an additional pathway. Together, these results suggest that the academic relevance of PCRI extends beyond the physical presence of counseling facilities to students’ psychological well-being, educational aspirations, and engagement with learning.

The psychological well-being and motivational channels identified here are likely to extend beyond the specific institutional setting of the CEPS sample, whereas the magnitude of the estimated PCRI coefficients is inherently context-dependent. Variation in educational policies, access to professional psychological services, students’ underlying support needs, and the availability of alternative support may affect the strength of these relationships. Accordingly, we view the mechanism results as evidence of broader psychological and motivational processes, while interpreting the estimated magnitudes within the 2014–2015 institutional context.

### 4.4 Moderating effects of PCRI

This section examines whether the relationship between psychological counseling room infrastructure (PCRI) and students’ academic performance varies across student and school contexts. Since PCRI captures both the availability and facility condition of school-based psychological counseling rooms, its academic benefits may be more salient for students who face greater psychological vulnerability or have fewer alternative sources of emotional and educational support. We therefore examine four moderators: students’ migration status, parental education level, teachers’ mental health training, and school location. To test these moderating effects, we estimate the following Equation (2):

$$\begin{array}{l} {\text{Grades}}_{i}=\alpha_{0}+\beta_{1}{\text{PCRI}}_{s}\times M+\beta_{2}{\text{PCRI}}_{s}+\beta_{3}M\\ +\alpha_{1}\sum {\text{Control}}_{i,h,t,s}+\sigma_{d}+\varepsilon_{i,h,t,s} \end{array}$$ (2)

where *M* denotes the moderator variable, including students’ migration status (Migrant), parental education level (Education), teachers’ mental health training (Trained), and school location (Rural). Other variables are the same as Equation 1. The coefficient of interest is $\beta_{1}$ , which captures whether each moderator strengthens or weakens the association between PCRI and students’ academic performance. A positive interaction coefficient indicates that the moderator strengthens the positive association between PCRI and academic performance, whereas a negative coefficient indicates that the moderator weakens this association. The results are reported in Table 6.

**Table 6:** Moderating effects.

Variables	(1)	(2)	(3)	(4)
	Students’ academic performance: SAP			
Migrant × PCRI	1.917*** (2.861)			
Education × PCRI		−0.310*** (−2.762)		
Trained × PCRI			−3.320*** (−6.521)	
Rural × PCRI				3.737*** (7.919)
PCRI	4.215*** (14.387)	5.414*** (10.514)	3.987*** (11.740)	6.124*** (14.920)
Control vars	Yes	Yes	Yes	Yes
County FE	Yes	Yes	Yes	Yes
Observations	6,055	6,018	6,055	6,055
Adj_*R*^2^	0.729	0.732	0.734	0.733

*** denotes significance at 1%, ** at 5%, and * at 10%. County FE is county fixed effects. Control Vars include student-level controls (Gender, PriorScore, InternetAccess, Disability, NumFriends), family-level controls (FamilyEco, CommunityEnv, LivingArrangement, ParentChildRel, Migrant, ParentalSupervision), classroom and homeroom-teacher controls (ClassSize, HT_Gender, HT_TeachingExp, HT_PsychTraining), and school-level controls (SchoolLocation, PublicSchool, StudentTeacherRatio, PrincipalTenure).

#### 4.4.1 Moderating role of students’ migration status

We first examine whether students’ migration status moderates the relationship between PCRI and academic performance. The variable Migrant equals 1 if the student is a migrant student and 0 otherwise. As shown in Column (1), the coefficient on Migrant × PCRI is positive and statistically significant, indicating that migrant status strengthens the positive association between PCRI and students’ academic performance. This result is consistent with a compensatory interpretation. Migrant students often face greater school adaptation pressure, disrupted peer networks, and weaker family or community support, which may increase their exposure to psychological stress and emotional adjustment difficulties (Free et al., 2014; Chen et al., 2019; Song et al., 2019). In this context, well-equipped psychological counseling rooms may provide an important institutional source of emotional support, adaptation guidance, and psychological security. By helping migrant students cope with stress and strengthen their sense of belonging in school, PCRI may produce larger academic benefits for this group.

#### 4.4.2 Moderating role of parental education

We next examine whether parental education moderates the relationship between PCRI and academic performance. Education is measured by the highest years of schooling completed by either parent. Column (2) shows that the coefficient on Education × PCRI is negative and statistically significant. This result indicates that higher parental education weakens the positive association between PCRI and academic performance. Conversely, PCRI has a stronger positive association with academic performance among students whose parents have lower educational attainment. This finding suggests that PCRI may compensate for limited family-based psychological and educational support. Students from less-educated families may receive less academic guidance, emotional support, and future-oriented educational planning at home. Prior research shows that lower family socioeconomic status and parental education are associated with greater risks of emotional and behavioral difficulties among children and adolescents (Bradley and Corwyn, 2002; Reiss, 2013). For these students, school-based psychological counseling infrastructure may help alleviate emotional barriers, strengthen academic confidence, and support learning engagement. Thus, PCRI may be especially beneficial for students from less-educated family backgrounds.

#### 4.4.3 Moderating role of teachers’ mental health training

We then examine whether teachers’ mental health training moderates the relationship between PCRI and academic performance. The variable Trained is measured by the number of core-subject teachers and head teachers who have received formal mental health training. Column (3) shows that the coefficient on Trained × PCRI is negative and statistically significant. This result indicates that teacher mental health training attenuates the positive association between PCRI and students’ academic performance. In other words, PCRI has a stronger academic benefit in schools where fewer teachers have received mental health training. This pattern is consistent with a substitution or compensatory mechanism. Teachers are often the first adults in schools to observe students’ emotional distress, behavioral changes, and learning difficulties. When teachers lack mental health training, students may have fewer opportunities to receive timely emotional support, early identification, or appropriate referral during daily classroom interactions. Prior studies show that teacher mental health literacy is important for identifying and supporting students with psychological needs, and that training can improve teachers’ knowledge, attitudes, and confidence in responding to student mental health problems (Kutcher et al., 2013; Ueda et al., 2021). Therefore, in schools with fewer mentally trained teachers, PCRI may become a more important institutional channel for addressing students’ unmet psychological needs, thereby generating stronger academic benefits.

#### 4.4.4 Moderating role of school location

Finally, we examine whether school location moderates the relationship between PCRI and academic performance. The variable Rural equals 1 if the school is located in a rural area and 0 otherwise. Column (4) shows that the coefficient on Rural × PCRI is positive and statistically significant, indicating that rural school location strengthens the positive association between PCRI and students’ academic performance. This result suggests that PCRI has greater compensatory value in resource-constrained school contexts. Rural schools often face shortages of professional support staff, weaker access to psychological services, and fewer comprehensive educational resources (Song, 2023; Graves et al., 2023). These constraints may leave rural students with fewer institutional channels for emotional regulation, stress management, and academic guidance. In such settings, the availability and quality of psychological counseling rooms may provide a particularly important source of psychological support and learning-related confidence. Therefore, PCRI may generate larger academic returns in rural schools because it helps compensate for broader gaps in school support resources.

Overall, the moderation analysis shows that PCRI has stronger academic benefits among students and schools with greater psychological support needs: migrant students, students from less-educated families, students in schools with fewer mentally trained teachers, and students in rural schools. These findings are internally consistent. They suggest that PCRI plays a compensatory role by providing institutionalized psychological support where students are more likely to experience psychological distress or where alternative support resources are relatively limited. Therefore, improving psychological counseling room infrastructure may contribute not only to student mental health, but also to educational equity by helping disadvantaged students translate psychological support into better academic outcomes.

### 4.5 Subject-specific effects of PCRI

To examine whether the positive relationship between PCRI and academic performance is confined to a particular subject, we re-estimate the baseline specification using Chinese, mathematics, and English scores as separate dependent variables. Table 7 reports the results. The coefficient on PCRI is positive and statistically significant across all three subjects, with estimates of 2.023 for Chinese, 4.339 for mathematics, and 7.040 for English. These results indicate that the positive association between PCRI and students’ academic performance is observed across language, quantitative, and foreign-language learning domains rather than being driven by a single subject.

**Table 7:** Subject-specific effects of PCRI.

Variables	(1)	(2)	(3)
	Chinese_score	Math_score	English_score
PCRI	2.023*** (8.188)	4.339*** (9.561)	7.040*** (18.151)
Control vars	Yes	Yes	Yes
County FE	Yes	Yes	Yes
Observations	6,055	6,055	6,055
Adj_*R*^2^	0.615	0.596	0.664

*** denotes significance at 1%, ** at 5%, and * at 10%. County FE is county fixed effects. Control Vars include student-level controls (Gender, PriorScore, InternetAccess, Disability, NumFriends), family-level controls (FamilyEco, CommunityEnv, LivingArrangement, ParentChildRel, Migrant, ParentalSupervision), classroom and homeroom-teacher controls (ClassSize, HT_Gender, HT_TeachingExp, HT_PsychTraining), and school-level controls (SchoolLocation, PublicSchool, StudentTeacherRatio, PrincipalTenure).

## 5 Conclusions and policy implications

### 5.1 Conclusion

Using nationally representative data from the 2014-2015 China Education Panel Survey (CEPS), this paper examines whether Psychological Counseling Room Infrastructure (PCRI) affects students’ academic performance. The results show that PCRI is positively and significantly associated with student achievement. Economically, a one-standard-deviation increase in PCRI is associated with a 3.26-point increase in academic performance, equivalent to approximately 4.92% of the sample mean. Mechanism analysis suggests that this effect operates through two channels: a psychological well-being channel, reflected in the improvement of students’ mental health and the enhancement of their confidence in the future, and a motivational enhancement channel, reflected in the boost of students’ educational self-expectations and the stimulation of learning engagement. Moderation analysis further shows that PCRI plays a stronger compensatory role among students and schools with greater psychological support needs, including migrant students, students from less-educated families, students in schools with fewer mentally trained teachers, and students in rural schools. Overall, these findings suggest that psychological counseling room infrastructure is not only a mental health resource, but also an academically meaningful and equity-enhancing form of school investment.

### 5.2 Policy implications

The findings suggest that school-based psychological support should be regarded as an integral component of the educational environment rather than merely as a remedial service for students who have already developed serious psychological difficulties. By supporting students’ emotional adjustment, future confidence, and engagement with learning, psychological support may also contribute to the broader conditions under which academic development takes place. Accordingly, policy attention should extend beyond the provision of counseling rooms toward the development of comprehensive psychological support systems that combine appropriate facilities, professionally trained personnel, teacher mental-health literacy, effective referral mechanisms, and, where appropriate, digital or remote support. The key policy objective is therefore not simply to expand physical infrastructure, but to ensure that psychological-support resources are accessible, functional, and effectively integrated into the everyday educational environment.

The stronger associations observed among migrant students, students from less-educated families, rural students, and students in schools with fewer psychologically trained teachers further suggest that psychological-support resources may be particularly valuable in disadvantaged settings. These students and schools may have more limited access to alternative sources of emotional, informational, and educational support outside the school environment. Education authorities may therefore consider differentiated resource allocation that prioritizes schools and student groups with greater psychological-support needs and weaker existing support capacity. In practice, this may involve strengthening professional staffing and teacher training in underserved schools, improving referral and support networks, and using remote or digital services to complement on-site resources where specialist personnel are scarce. Such targeted provision may help psychological-support systems contribute not only to student well-being, but also to educational equity.

### 5.3 Temporal limitations and future research

An important limitation of this study concerns the temporal scope of the data. The CEPS observations were collected in 2014-2015, before the COVID-19 pandemic, the “Double Reduction” reform, and the subsequent expansion and diversification of school-based mental-health services. Changes in educational policy, students’ psychological needs, and the increasing use of digital and technology-assisted forms of support may have altered both the availability and the role of psychological counseling infrastructure. The magnitude of the estimated association between PCRI and student outcomes should therefore be interpreted within the institutional context of the study period rather than mechanically extrapolated to contemporary schools.

External validity also warrants caution. Our analytical sample consists of eighth-grade students in Chinese junior middle schools, and the results may not generalize directly to students at earlier or later stages of schooling, for whom psychological needs, academic pressure, and school-support systems may differ. Likewise, institutional differences in counseling provision, school governance, and educational resources may limit direct generalization to other countries. At the same time, the 2014-2015 setting provides useful historical baseline evidence from a period when psychological counseling infrastructure remained relatively heterogeneous across Chinese middle schools. Future research using more recent cohorts, other grade levels, and comparable data from different educational systems could examine the extent to which the relationships identified here persist across institutional and developmental contexts.

A further limitation concerns measurement. PCRI captures only the observable institutional and infrastructural dimension of psychosocial infrastructure and should not be interpreted as a comprehensive measure of the broader psychosocial environment, shared norms, relational climate, or school culture. More specifically, the measure reflects the availability and reported condition of counseling-room infrastructure, but does not provide information on actual service utilization, counselor qualifications, service intensity, or student satisfaction. Similarly, the mental-health measure relies on students’ recent self-reports, while learning engagement is a behavioral proxy rather than a direct measure of intrinsic motivation. Future research combining infrastructure information with objective service records and student-level utilization data would allow a more comprehensive assessment of the quality and effectiveness of school-based psychological support.

Although restricting the sample to schools with confirmed random classroom assignment reduces concerns about student sorting across classrooms within schools, it does not eliminate potential sorting across schools. Families may select schools based on educational quality, location, reputation, or other characteristics that may also be correlated with psychological-support infrastructure. Our observable school-level comparisons alleviate, but cannot fully resolve, this concern.

## Data Availability

The original contributions presented in the study are included in the article/Supplementary material, further inquiries can be directed to the corresponding author.

## Footnotes

1 The ten problems include: (1) frustration; (2) being too depressed to concentrate on things; (3) unhappiness; (4) life is not interesting; (5) not being able to get up the energy to do things; (6) sadness, grief; (7) nervousness; (8) worrying too much; (9) having a premonition that something bad is going to happen; and (10) being too energetic and not paying attention in class.
