# Supplementary material for: The hidden classroom: how psychosocial infrastructure shapes academic achievement and educational equity in China
Source: Front Psychol. 2026 Oct 7;17:1902392. doi: 10.3389/fpsyg.2026.1902392 (PMC13685429; doi:10.3389/fpsyg.2026.1902392)
Supplement: Supplementary file 1 [file Supplementary_file_1.docx]

**Appendix**

**Table A.1. Sample Selection Procedure**

| **Sample selection criterion** | **Observations excluded** | **Remaining observations** |
| --- | --- | --- |
| Initial CEPS baseline sample, 2013–2014 | — | 19,487 |
| Exclude students who were not in Grade 7 at baseline | 9,208 | 10,279 |
| Exclude Grade-7 students who were not successfully followed in 2014–2015 | 359 | 9,920 |
| Exclude observations that could not be matched with the required principal, classroom, and parent questionnaire information | 1,653 | 8,267 |
| Exclude students in schools without confirmed random classroom assignment | 2,165 | 6,102 |
| Exclude observations with missing PCRI | 47 | 6,055 |
| Exclude observations with missing baseline covariates | 0 | **6,055** |

Table A.2. Observable School Characteristics: Random-Assignment Schools versus the Full CEPS School Sample

| Variables | Mean_Random | Mean_Full | Diff | p-value | Smd |
| --- | --- | --- | --- | --- | --- |
| School district characteristics | | | | | |
| PCRI | 1.295 | 1.176 | 0.119 | 0.532 | 0.174 |
| SchoolLocation | 0.455 | 0.529 | -0.075 | 0.575 | -0.147 |
| PublicSchool | 0.909 | 1.000 | -0.091 | 0.199 | -0.445 |
| StudentTeacherRatio | 12.904 | 11.150 | 1.754 | 0.149 | 0.440 |
| PrincipalTenure | 11.443 | 9.118 | 2.326 | 0.173 | 0.417 |
| Classroom teacher characteristics | | | | | |
| ClassSize | 3.855 | 3.851 | 0.004 | 0.936 | 0.016 |
| HT_Gender | 0.636 | 0.667 | -0.030 | 0.716 | -0.063 |
| HT_TeachingExp | 14.597 | 18.538 | -3.941 | 0.007 | -0.513 |
| HT_PsychTraining | 0.199 | 0.436 | -0.237 | 0.001 | -0.521 |
| Family and parent characteristics | | | | | |
| FamilyEco | 2.750 | 2.529 | 0.221 | 0.256 | 0.321 |
| CommunityEnv | 0.886 | 0.941 | -0.055 | 0.504 | -0.193 |
| LivingArrangement | 0.670 | 0.824 | -0.153 | 0.213 | -0.352 |
| ParentChildRel | 0.489 | 0.353 | 0.136 | 0.309 | 0.273 |
| Migrant | 0.284 | 0.294 | -0.010 | 0.934 | -0.022 |
| ParentalSupervision | 13.852 | 14.353 | -0.501 | 0.471 | -0.201 |

Note: This table compares observable school-level characteristics between schools with confirmed random classroom assignment and the full CEPS school sample. “Difference” denotes the difference in group means, and SMD denotes the standardized mean difference. The absence of statistically significant differences across these characteristics suggests that observable school-level selection is unlikely to materially affect the sample composition.

**Table A.3 Variable definitions**

| Variables | Definitions |
| --- | --- |
| Student characteristics variables | |
| SAP | See section 3.3.2 |
| Gender | 1 for men, otherwise 0 |
| PriorScore | Mean scores of students' standardized scores in language arts, mathematics, and English in the 13-14 school year in the questionnaire |
| InternetAccess | 1 for students who have access to the Internet and otherwise 0 |
| Disability | 1 for students with physical disabilities, otherwise 0. |
| NumFriends | logarithmic value of the number of best friends filled by the students in the questionnaire |
| Family and parent characteristics variables | |
| FamilyEco | According to the parents' self-assessment of the family's economic conditions, the family was categorized as very difficult, relatively difficult, moderate, relatively wealthy and very wealthy with values ranging from 1 to 5, respectively. |
| CommunityEnv | 1 for identify communities with minor pollution and no pollution as environmentally sound, 0 for communities with very serious pollution and more serious pollution |
| LivingArrangement | 1 for students living with both parents, otherwise 0 |
| ParentChildRel | 0 for students who answer that they are not close to either parent, otherwise 1 |
| Migrant | 1 if the student is a migrant and 0 if the student is a local student |
| ParentalSupervision | Based on the parents' supervisory attitudes towards the six issues in learning and life, assign a value of 1 to “don't care”, a value of 2 to “don't control too much” and a value of 3 to “don't control too much”, and sum up the scores. |
| Classroom teacher characteristics variable | |
| ClassSize | Logarithmic value of the planned number of classes in the school |
| HT_Gender | 1 for classroom teacher is men, otherwise 0 |
| HT_TeachingExp | Years of teaching career of the classroom teacher |
| HT_PsychTraining | 1 for the classroom teacher having mental health training, otherwise 0 |
| School district characteristics variables | |
| PCRI | See section 3.3.1 |
| SchoolLocation | 1 for schools located in urban areas of cities/counties, otherwise 0 |
| PublicSchool | 1 for schools in the questionnaire that are public schools, otherwise 0 |
| StudentTeacherRatio | Number of students/number of teachers |
| PrincipalTenure | Principal's tenure at the school |

**Table A.4 Alternative Constructions of Students’ Academic Performance**

| Variables | Students’ academic performance: SAP | |
| --- | --- | --- |
|  | (1)z-scores | (2)First principal component |
| PCRI | 0.201*** | 0.349*** |
|  | (14.679) | (14.717) |
| Control Vars | YES | YES |
| County FE | YES | YES |
| Observations | 6055 | 6055 |
| Adj_R2 | 0.733 | 0.733 |

Notes: Same as Table 2.

**Table A.5. Multicategorical Specification of Psychological Counseling Room Infrastructure**

| Variables | Students’ academic performance: SAP |
| --- | --- |
|  | (1) |
| PCRI_medium | 1.312** |
|  | (2.276) |
| PCRI_good | 7.628*** |
|  | (12.204) |
| Control Vars | YES |
| County FE | YES |
| Observations | 6055 |
| Adj_R2 | 0.732 |
| PCRI_good−PCRI_medium | 6.315*** |

Notes: PCRI_medium equals 1 for schools with a psychological counseling room whose facilities need improvement (PCRI=1), while PCRI_good equals 1 for schools with a psychological counseling room in good condition (PCRI=2). Schools without a psychological counseling room (PCRI=0) constitute the reference category. The final row reports the test of equality between the coefficients on PCRI_good and PCRI_medium. All specifications include the baseline control variables and county fixed effects. t-statistics are reported in parentheses. ***, **, and * denote significance at the 1%, 5%, and 10% levels, respectively.

**Appendix Table A.6. Sensitivity to Selection on Unobservables**

|  | Short specification | Full specification |
| --- | --- | --- |
| Variables | Students’ academic performance: SAP | |
| PCRI | 4.738*** | 4.467*** |
|  | (16.378) | (15.653) |
| Individual-level controls | YES | YES |
| Other controls | NO | YES |
| R² | 0.677 | 0.730 |
| Oster δ | 1.763 | 1.867 |

Notes: Same as Table 2.

**Table A.7 Inverse-Probability-of-Selection-Weighted Estimates**

| **Variable** | **(1) Baseline** | **(2) IPSW** |
| --- | --- | --- |
| PCRI | 4.467*** | 4.260*** |
|  | (15.653) | (4.551) |
| Controls | Yes | Yes |
| County FE | Yes | Yes |
| Observations | 6,055 | 6,055 |
| R-squared | 0.732 | 0.734 |

Notes: This table reports estimates using inverse-probability-of-selection weighting. We first estimate the probability that an observation belongs to a school with confirmed random classroom assignment based on observable student-, family-, classroom/teacher-, and school-level characteristics. The inverse of the predicted selection probability is then used to reweight observations in the restricted sample. Column (1) reports the baseline estimate, and Column (2) reports the IPSW estimate. All specifications include the same set of control variables and county fixed effects as the baseline model. Standard errors, reported in parentheses, are clustered at the school level. We also construct stabilized selection weights and trim extreme weights; the resulting estimates are identical to those reported in Column (2) and are therefore not separately tabulated. ***, **, and * denote statistical significance at the 1%, 5%, and 10% levels, respectively.
